# Pulmonary Cryptococcosis With Rapid Growth on Positron Emission Tomography/Computed Tomography

**DOI:** 10.1002/rcr2.70322

**Published:** 2025-08-21

**Authors:** Yosuke Fukuda, Naruhito Oda, Hironori Sagara, Akihiko Tanaka

**Affiliations:** ^1^ Department of Medicine, Division of Respiratory Medicine Yamanashi Red Cross Hospital Fujikawaguchiko‐machi Yamanashi Japan; ^2^ Department of Medicine, Division of Respiratory Medicine and Allergology Showa Medical University School of Medicine Shinagawa‐ku Tokyo Japan

**Keywords:** immunosuppression, positron emission tomography/computed tomography, pulmonary cryptococcosis, rheumatoid arthritis

## Abstract

A 64‐year‐old woman with rheumatoid arthritis and asthma developed a rapid growth and uptake of ^18^F‐fluorodeoxyglucose (FDG)‐avid lung nodule. Despite suspicion of malignancy, the biopsy confirmed pulmonary cryptococcosis (PC). Regardless of FDG uptake, PC should be considered in the differential diagnosis.

## Graphical abstract

A 64‐year‐old woman with rheumatoid arthritis and asthma developed a rapid growth and uptake of 18F‐fluorodeoxyglucose (FDG)‐avid lung nodule. The biopsy confirmed pulmonary cryptococcosis (PC). Regardless of FDG uptake, PC should be considered in the differential diagnosis.

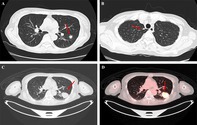

A 64‐year‐old non‐smoker woman with a 6‐year history of rheumatoid arthritis, who had recently transitioned from methotrexate to adalimumab, presented with a 4‐week history of persistent cough. She was newly diagnosed with asthma and began therapy, including inhaled fluticasone furoate (100 μg once daily). Concurrently, chest computed tomography (CT) revealed a 12‐mm nodule in the left S6 segment along with several smaller nodules (Figure 1A,B). We performed transbronchial lung biopsies (TBLB), which did not reveal a definitive diagnosis. Two months later, positron emission tomography/computed tomography (PET/CT) showed nodule enlargement to 36 mm (Figure 1C) and intense uptake of ^18^F‐fluorodeoxyglucose (^18^F‐FDG), with a maximum standardised uptake value (SUVmax) of 17.97 (Figure 1D). A second TBLB revealed necrotic tissue containing numerous capsule‐like organisms (Figure 2A), which stained positively on fungal‐specific histochemical stains (Figure 2B–D), confirming pulmonary cryptococcosis (PC). We attributed disease onset and progression to immunosuppression associated with tumour necrosis factor‐α inhibition and inhaled corticosteroid use [1]. In immunocompromised patients, PC often manifests in the subpleural region, with a median SUVmax of six on PET/CT [2]. Despite intense FDG uptake, PC should remain in the differential diagnosis for immunosuppressed individuals, highlighting the importance of tissue biopsy for definitive diagnosis.

**FIGURE 1:**
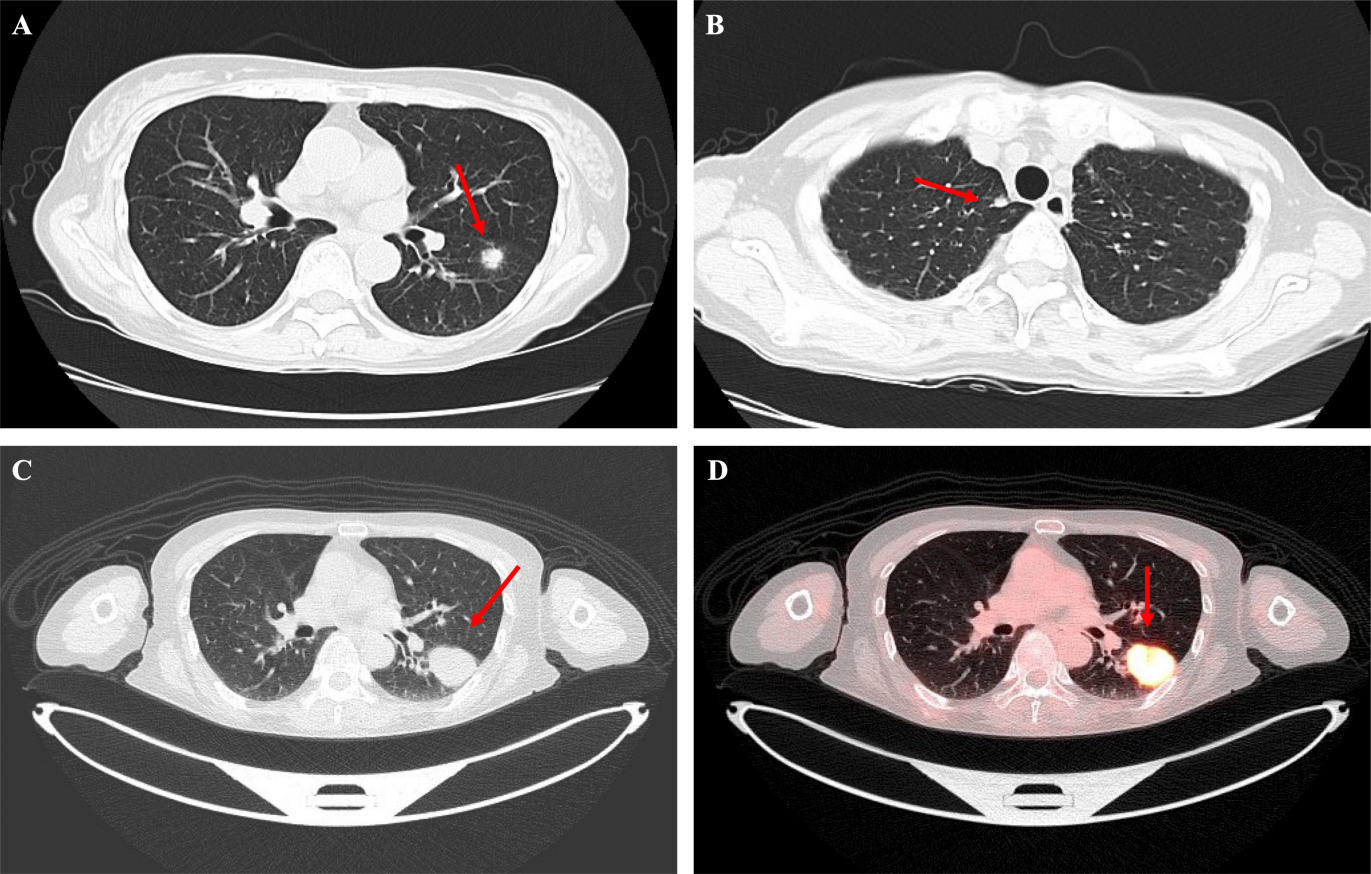
Chest computed tomography (CT) reveals a 12‐mm nodule in the left S6 segment (A) and multiple smaller nodules (B) (red arrows). Positron emission tomography/CT shows enlargement of the nodule to 36 mm (C), with high 18F‐fluorodeoxyglucose uptake (standardised uptake value max, 17.97) (D) (red arrows).

**FIGURE 2:**
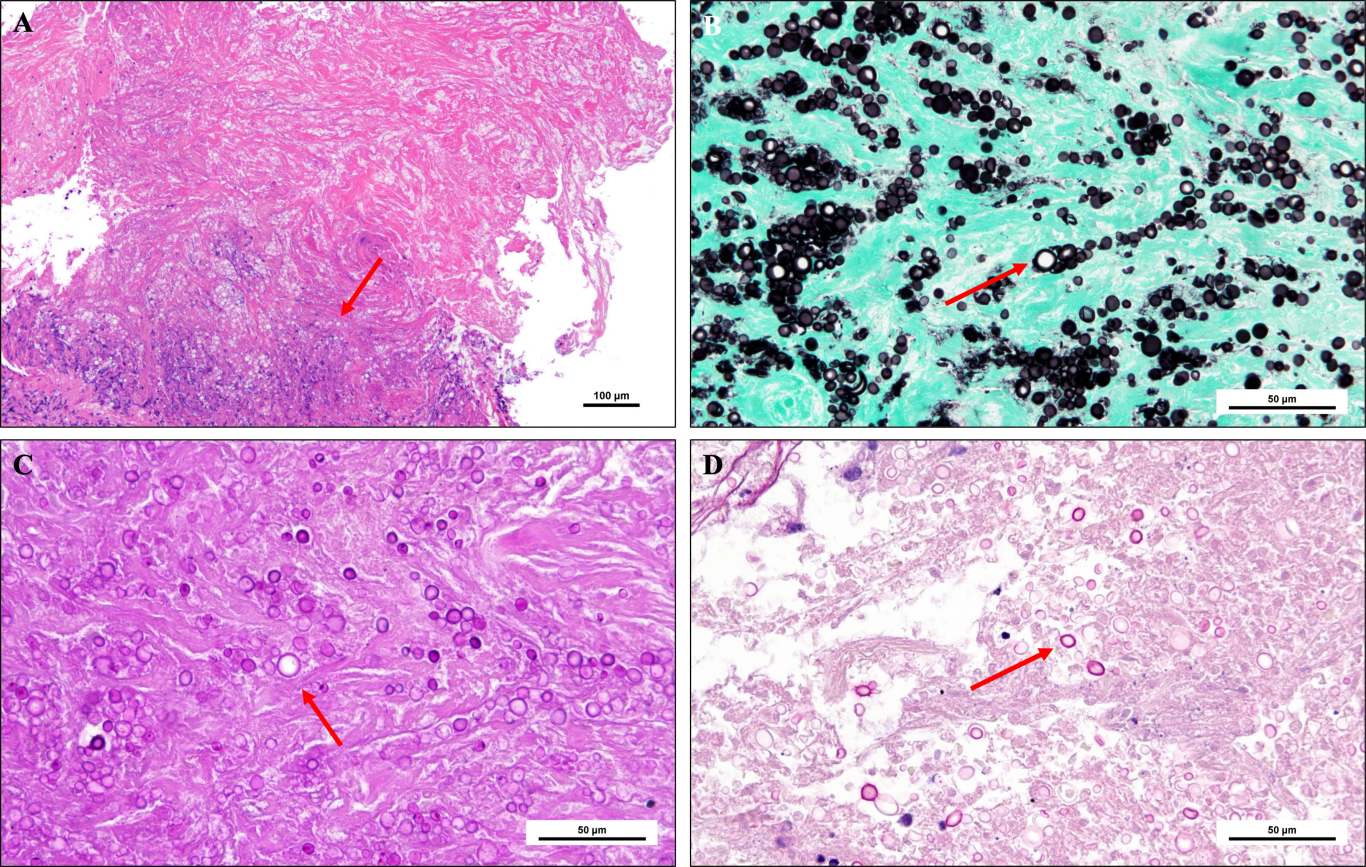
Transbronchial lung biopsy of left lower lobe nodule. (A) Necrotic tissue containing numerous capsule‐like organisms (haematoxylin–eosin stain, magnification ×10; red arrow); (B) The capsule stains reddish‐purple (periodic acid–Schiff stain, ×40; red arrow); (C) The organisms exhibit black‐stained cell walls (Grocott's methenamine silver stain, red arrow); (D) The capsule stains reddish (mucicarmine stain, ×4; red arrow).

## Author Contributions

Conception: Y.F., N.O.; Design of the work: Y.F.; Acquisition, analysis or interpretation of data for the work: Y.F., N.O., A.T., H.S.; Drafting the work: Y.F., N.O.; Revising it critically for important intellectual content: A.T., H.S.; Final approval of the version to be published: Y.F., N.O., A.T., H.S.

## Consent

The authors declare that written informed consent was obtained for the publication of this manuscript and accompanying images using the consent form provided by the Journal.

## Conflicts of Interest

The authors declare no conflicts of interest.

## Data Availability

Data sharing not applicable to this article as no datasets were generated or analysed during the current study.
